# Aid to a Declining Matriarch in the Giant Otter (*Pteronura brasiliensis*)

**DOI:** 10.1371/journal.pone.0011385

**Published:** 2010-06-30

**Authors:** Lisa C. Davenport

**Affiliations:** Center for Tropical Conservation, Duke University, Durham, North Carolina, United States of America; University of Sussex, United Kingdom

## Abstract

Scientists are increasingly revealing the commonalities between the intellectual, emotional and moral capacities of animals and humans. Providing assistance to elderly and ailing family members is a human trait rarely documented for wild animals, other than anecdotal accounts. Here I report observations of multiple forms of assistance to the declining matriarch of a habituated group of giant otters (*Pteronura brasiliensis*) in Manu National Park, Peru. The otter group had been observed annually for several years and all members were known individually. In 2007, the breeding female of the group failed to reproduce and appeared to be in physical decline. She begged from other family members 43 times over 41 contact hours and received food 11 times. Comparisons with 2004–2006 demonstrate that the family's behavior in 2007 constitutes a role-reversal, in which the majority of assistance and prey transfers accrued from young-to-old rather than from old-to-young. As in human societies, both non-adaptive and adaptive hypotheses could explain the family members' aid to their declining matriarch. I suggest that giant otter families may benefit from the knowledge and experience of an elderly matriarch and “grandparent helper,” consistent with the “Grandmother Hypothesis” of adaptive menopause in women.

## Introduction

Human exceptionalism – the belief that humans are unique in the living world, particularly in behavioral, emotional, and moral capacities – still claims many advocates [1] despite the fact that a number of behaviors once considered unique to humans have been found in other animals. For example, tool use, once thought the quintessential human trait, is now known for some birds [2] and primates [3], and the transmission of cultural practices has been reported in cetacean [4] and primate [5], [6] societies. Capuchin monkeys [7] and dogs [8] refuse to participate in reward schemes that provide unequal pay for equal effort, suggesting a recognition and rejection of inequity (but see [9]). Finally, menopause, the age-specific, permanent cessation of reproduction in women [10], is thought to also function in matrilineal cetaceans such as short-finned pilot whales (*Globicephala macrorhynchus*), killer whales (*Orcinus orca*), and probably others such as sperm whales (*Physeter macrocephalus*) [11], [12].

A human behavior that is rarely documented in animals is long-term assistance to elderly family members. Anecdotal reports have appeared previously describing events in which sick or injured family members received short-term assistance, particularly in cooperative species such as African Elephants (*Loxodonta africana*) [13]–[14], African Wild Dogs (*Lycaon pictus*) [15], Mountain Gorillas (*Gorilla gorilla berengei*) [16] and Bonobos (*Pan paniscus*) [17]. However, to my knowledge, no other study has reported details of ongoing assistance to aid a declining elder in a family of wild animals, in this case including prey-sharing over an extended period of time.

### The Giant Otter

The giant otter is unique among the 13 extant species of otters in breeding cooperatively [18]. The mated pair remains together year-round, and is believed to be monogamous [18]. The young stay with the family in which they were born for 1.5 to 4 y [18]–[21]. Reproductive maturity occurs around 2.5–3 y [22], and the large family size (typically 4–10 individuals), engenders complex intra-familial social interactions [18], [19], [21]. All family members assist young through various tasks such as defense, grooming, and sharing prey, although the relative contribution of helpers varies by individual and by age [19]. The main prey of giant otters is fish [18], but in Manu National Park, occasionally other prey items are eaten, including juvenile black caiman, bivalves, and frogs [19]. Young cubs and juveniles (up to ∼1 y) rely heavily on food subsidies obtained from their parents and siblings through vociferous begging.

### The Cocha Salvador Family

Fifteen animals were observed on Cocha (lake) Salvador between 2004–2007 (206 total contact hours), including 12 juveniles and 3 breeding adults. Cacao, the matriarch, was first seen in Salvador in 1998 with offspring believed to have been born the previous year [23]. She and her first partner, Fantasma, produced litters of 1–4 cubs annually through to 2006 [23]. In September 2007, Fantasma was absent from the Cocha Salvador group, presumably having died after I last observed him in October 2006. A photograph of Fantasma as a subadult (∼1 y) taken at Cocha Cashu in 1992 indicates that he was born in 1991 [24]. He thus died at approximately 15 y of age, the longest recorded age for a wild giant otter [23]. In 2007, a new adult male appeared with Cacao (Firecat, age unknown). Three of Cacao's offspring from previous years remained in the family: Ziggy (female, born 2004), Achilles (male, born 2005), and Caiman (male, born 2006). As no female under 3 y of age has ever been observed with a litter [23], based on the 1997 litter, a minimum age for Cacao in 2007 is 13 y.

## Results

**Figure 1:**
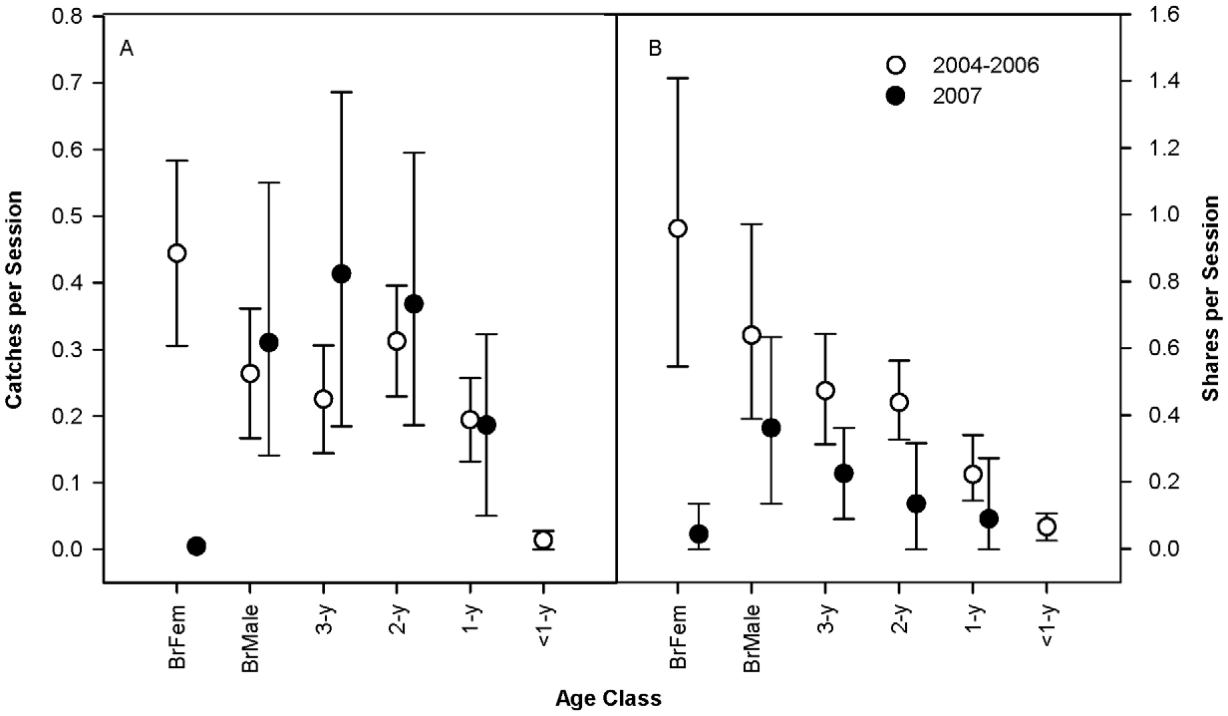
Bootstrapped mean and 95% confidence intervals on average counts per 3-h session by age class of A) catches of large (≥30 cm) fish; and, B) prey sharing (prey of all sizes), comparing 2004–2006 vs. 2007 observations. Data represent 15 animals, including 12 juveniles and 3 breeding adults. The age class “BrFem” denotes data on the breeding female, Cacao. No cubs <1 y were present in 2007.

Prior to 2007, Cacao acted as a leader and top provisioner within the Cocha Salvador family [19]. She coordinated group movements and social interactions, being often observed to initiate hunting bouts or changes in locations, and she exceeded other family members in her rates of catching large fish (≥30 cm) and sharing prey (Table 1 and Figure 1). She was never seen to beg in 2004–2006, although she did receive unsolicited donations on 2 occasions when exceptionally large prey items were being shared around the entire group.

**Table 1:** Outcomes of all begging bouts between potential donors and beggars by year and potential donor.

							Donor							*Totals*
	Otter	Cacao	Fantasma	Rambo	Ziggy	Achilles	unk	Diabolo	Frita	Virute	Fantasmita	Mars	Saguarito	
	Year born	≤1994	1991	2004	2004	2005		2002	2002	2002	2002	2003	2003	
2004	**Share**	38	31	1	1	-	90	13	11	13	14	8	9	***229***
	**No Share**	8	13	4	4	-	12	10	15	7	11	10	4	***98***
	**Steal**	2	3	0	4	-	14	1	4	5	2	0	0	***35***
	***2004 Total***	***48***	***47***	***5***	***9***	***-***	***116***	***24***	***30***	***25***	***27***	***18***	***13***	***362***
2005	**Share**	15	10	2	2	1	35	-	-	15	14	8	7	***109***
	**No Share**	7	2	9	5	0	3	-	-	5	7	6	7	***51***
	**Steal**	1	0	0	0	0	2	-	-	2	2	1	0	***8***
	***2005 Total***	***23***	***12***	***11***	***7***	***1***	***40***	***-***	***-***	***22***	***23***	***15***	***14***	***168***
2006	**Share**	18	6	7	14	6	30	-	-	-	-	-	-	***81***
	**No Share**	8	4	9	5	2	5	-	-	-	-	-	-	***33***
	**Steal**	0	0	1	0	0	1	-	-	-	-	-	-	***2***
***2006 Total***		***26***	***10***	***17***	***19***	***8***	***36***	***-***	***-***	***-***	***-***	***-***	***-***	***116***

Outcomes are scored as a “share” “no share” (refusal to share) or “steal” as described in text. Cacao and Fantasma were the breeding female and male during 2004–2006, while all others are their offspring. Year of birth given where known. Animals to the right of the unknown (“unk”) donor column are juveniles who dispersed before the 2006 field season. Observations are based on 86, 45, and 34 contact hours in 2004, 2005, and 2006, respectively.

In 2007, Cacao produced no young, and her eyesight and mobility appeared compromised. She required assistance from her 4 family members in at least two contexts, both novel behaviors for a former leader and matriarch. First, Cacao occasionally became separated from the group when they engaged in fast chases after a school of fish without her. On three such occasions, she had to employ the loud “wavering scream” [18] to signal her distress, whereupon other animals assisted her to rejoin the group by swimming to her and directing her to the group's location.

Second, while Cacao caught medium and small fish, she failed to catch even one large fish in 2007 (Figure 1); presumably to compensate, she begged from family members for portions of prey items (Figure S1). In contrast to young otters who vocalize loudly and continuously before grabbing or receiving food, Cacao typically waited quietly in front of a family member in possession of a large prey item, squinting and staring at the other otter for many minutes. While waiting for a share, she vocalized infrequently or just immediately prior to approaching the potential donor to acquire the remainder of the prey item (Video S1). During the 41 contact hours I observed the group in 2007, Cacao begged from others 43 times, receiving 11 shares (Table 2). The next most frequent beggar, Caiman, a 1 y male, begged 7 times in the same period and received 6 shares. All family members but Achilles, a 2 y male, shared with Cacao in response to her begging, although Cacao's begging success (26% of begs resulted in a share) was lower than that of juveniles in previous years. Begging success for juveniles in 2004–2006 measured 63%, 65%, and 70% in each year, respectively (Table 1).

**Table 2:** Matrix of counts of shares|no shares observed between all beggars and potential donors, September 2007.

			Potential Donor						
Beggar		Achilles	Cacao	Caiman	Firecat	Ziggy	unk	*TOTAL Shares Rec'd*	*TOTAL Begs*
	Achilles	-	0|0	0|0	0|1	0|0	0|0	*0*	***1***
	Cacao	0|7	-	3|8	4|3	3|13	1|1	*11*	***43***
	Caiman	1|0	1|0	-	1|0	1|1	2|0	*6*	***7***
	Firecat	1|1	0|0	0|0	-	1|0	0|0	*2*	***3***
	Ziggy	0|0	0|0	0|0	0|0	-	0|0	*0*	***0***
***TOTAL Shares Given***		***2***	***1***	***3***	***5***	***5***	***3***	***19***	
***TOTAL Potential Shares***		***10***	***1***	***11***	***9***	***19***	***4***		***54***

Based on 41 hours of observation.

## Discussion

Cacao's failure to catch large fish, frequent begging, and need for assistance in staying with the group in 2007 are observations that contrast dramatically with her role as provider and leader in previous years. Comparisons between the two periods suggest that the family's behavior in 2007 constitutes a role-reversal in which the majority of assistance and prey transfers accrued from young-to-old rather than from old-to-young. That Cacao's family members regularly assisted her, including sharing prey is, to my knowledge, a new observation for the species. It is also of considerable general interest given that assistance to elderly or injured animals is so seldom reported for wild animals.

Figure 1 demonstrates the 2007 declines in Cacao's rates of catching large (≥30cm) fish and sharing prey, but it also shows that generally, sharing rates for other family members decreased in 2007 relative to 2004–2006, while catch rates of large fish increased (but rarely with statistical significance). The latter observations likely reflect the absence of young cubs. Juveniles <1 y beg considerably more than animals of older ages, and when hunting with young cubs (<6 mo), giant otters specialize on smaller prey items [19]. Cacao's family members may also have increased catch rates to replace the lost subsidy she provided previously, although these data are inconclusive.

In the only other instance in which I observed an incapacitated giant otter, the individual was a 2 y male that suffered a double fracture in his front leg after a fight with a black caiman (*Melanosuchus niger*) at Cocha Cashu. The injured male retired to the family den, while the rest of the group moved to another den off the lake. He emerged after 3 days to hunt with some success, but observations ended about 1 week later. He was not seen the following year. This contrast in the response of the family group to an injured member suggests that Cacao's identity as the family matriarch may be of consequence.

Assistance to a senescing matriarch might reflect misdirected (non-adaptive) behaviors that accrue no long-term or indirect fitness benefits to those helping. In the Salvador family, assisting Cacao might represent helping behavior that would otherwise be directed to young of the year, but in their absence was redirected to an older individual. Since yearlings are still begging when new young arrive, giant otter helpers are well-accustomed to year-round food sharing. Assistance might also reflect a strong familial attachment or conditioning to obey a high-ranking group member. Adult chimpanzees of both sexes have been observed to come to the aid of their mother, even when no longer in need of her protection [25]. Some social carnivores (e.g. wolves [26], [27]) allocate food according to a dominance hierarchy, with young forced on occasion to give food to more dominant older animals. A dominance hierarchy determining food sharing has never been observed in giant otters, however [21], and Cacao's low begging success compared to juveniles seems to argue against assistance being motivated by a hierarchical claim. Cacao's relative restraint in vocalizing, compared to juveniles, perhaps explains the discrepancy.

Alternatively, assistance to a senescing mother might be adaptive, providing indirect fitness benefits to existing and/or future offspring of all family members [28]. Greve et al. [29] have recently suggested that investment in elderly family members or unrelated associates (“senators”) who harbor special knowledge and experience will be evolutionarily selected in animal societies where certain preconditions exist. These preconditions include longevity, extended adolescence, communication skills, cooperation, and a variable habitat [29]. The case of the giant otter meets all these hypothesized preconditions, and additionally, giant otters experience low mortality among adult territory-holders, contending with few natural predators [30]. In giant otters, as in elephants [11], [31]–[34], a non-reproductive matriarch and “grandmother helper” [35] could continue to provide long-lived offspring and grandchildren with key benefits to survival, such as training in hunting and social development, social knowledge of seasonal locations of food and shelter, or allomothering services such as babysitting [31]–[34]. This suggestion would be consistent with the “grandmother hypothesis” of adaptive menopause [36]–[39] and is supported by prior observations in both wild [40] and captive [22], [41]–[42] giant otter families.

In the only other long-term demographic study published on giant otters (also in the Manú Biosphere Reserve), Groenendijk and Hayek report multiple cases of a sister or daughter of a breeding female acquiring the breeding position with immigrant males, occasionally with the previous breeding female assisting as helper [40]. Of particular note, in 2001 at Cocha Otorongo (also in the Manu National Park), the breeding female Isla stopped reproducing at age 10 after 3 years with a new male, after which her daughter Microbio took over the breeding spot with her step-father. The 10 y old Isla stayed with the family, but specifics of her helping behaviors as a grandmother-helper were not obtained [40].

Evidence from captive breeding also suggests that menopause may limit reproduction in female giant otters around 10–12 y of age [22], [41], [42]. Sykes-Gatz [22] first compiled data on reproductive cessation in captive giant otters. In captivity, individuals of both sexes typically live to 16–19 y [42], and the oldest age for a captive female giant otter is 19 y [22]; however, to my knowledge, no females have bred successfully beyond ∼11 y. The oldest published age at reproduction comes from the Cali, Colombia Zoo. One breeding female there produced nine litters between 1999 and 2004, ending reproduction in 2004 at ∼11 y of age, after a period of increasingly infrequent estrus and mating periods [41]. As of this writing (2009), she was still alive in the colony, but non-reproductive [42]. In the Dortmund Zoo (Germany), a female survived but ceased estrus after a difficult pregnancy at 9 y 4 mo [22], and in the Hagenbeck Tierpark (Germany), a female died at 11 y after a uterine infection during pregnancy [22]. Giant otter males do not appear to experience such early reproductive declines [22]–[24], [42]. In the wild, Fantasma (Cacao's first partner) bred at 15 y [24]. In captivity, males have bred at 14 y of age [22].

These data suggest that female giant otters may experience a post-reproductive period of life, possibly indicative of menopause, of considerable length, in which they may continue to assist female relatives in raising young. If true, giant otters would join cetaceans as one of the few other mammals besides humans thought to undergo menopause [11], [12]. The ability to habituate and observe giant otters at close range could allow detailed behavioral observations to give us a better understanding than for matrilineal cetaceans of the helping roles that such grandmother-helpers provide their grandchildren and older offspring.

Cooperative breeders like giant otters might be expected to experience lower selection for reproductive senescence and menopause, given that adults receive help in rearing young, theoretically lowering the cost of reproduction to aging females. However, in giant otters, the breeding female bears a disproportionate burden in bearing and raising the young in their first months of life. The cubs do not leave the den for the first 5 weeks of life, and they continue to nurse for up to 5 mo [20]. When lactating, the female must produce milk exclusively from her own hunting efforts; in over 500 h observing two families of otters, I never saw a female with young cubs beg for food or have it brought to her or the cubs inside the den. Only once the cubs can swim and follow the family on hunting bouts do they receive significant food subsidies from helpers. In the meerkat (*Suricata suricatta*), another cooperative breeder that undergoes reproductive senescence, the number of pups that survived to emerge from the den declined with the age of the breeding female; however, the number of emerged pups that survive to independence did not relate to her age [43]. Cooperative breeding, therefore, does not seem to preclude an adaptive basis for reproductive senescence and/or menopause in females of certain species where high costs of reproduction accrue disproportionately to the female during and soon after parturition. Rather, as suggested in [29], [31], sociality and cooperation may increase the value of older family members in cooperative species, where experience and social information-sharing furthers group survival.

In July 2008, Cacao was absent from the group and her oldest daughter Ziggy had assumed the role of breeding female. While Cacao's potential role as a “grandmother helper” was therefore not realized in this instance, I suggest that it is nevertheless possible that post-menopausal matriarchs may be a valuable resource for Giant Otter families, providing benefits that motivate offspring to assist them. Long-term demographic and behavioral studies should help clarify the significance of these rare and fortuitous observations.

## Methods

### Ethics Statement

Data collection methods were approved on an annual basis by officials at INRENA, the Peruvian Natural Resources Institute that oversees management of protected areas (recently renamed SERNANP under the Ministry of the Environment). In addition, officials of Manu National Park approved the research methodology annually, and received annual reports. Giant otters can be recognized individually throughout their lives by unique white markings on their throats (Figure S2), so no capture or marking of animals was undertaken for any purpose. The USDA Animal Welfare Act exempts purely observational studies of this type from oversight, but guidelines established by the Animal Behavior Society [44] and American Society of Mammalogists [45] are also relevant, specifically in recommending the use of a minimal number of individuals for any study, and considering the potential harm from strictly observational work. In this study, I determined that the main potential impact to the wild otter family was habituation to people, which could affect their long-term survival if habituated animals dispersed outside the park boundaries. However, as the animals at Cocha Salvador are also observed almost daily by tourist boats during the dry season, the animals were already habituated to close observation at the start of the study, and the additional impact of this study was deemed to be negligible.

### Study Site

The Manú Biosphere Reserve (MBR), Peru, protects the entire watershed of the Río Manú, and maintains Peru's best populations of such rare animals as giant otter (*Pteronura brasiliensis*), black caiman (*Melanosuchus niger*) and Orinoco goose (*Neochen jubata*) [19]. The lowland region of the reserve is dominated by tropical moist forest, with annual rainfall of ∼200 cm and elevation ∼325 m [46], [47].

Periodic channel avulsion creates numerous isolated oxbow lakes along the Río Manú's main course [48], [49]. As nutrient sinks, these lakes are highly productive habitats, and are preferred core territories for giant otters [20]. Cocha Salvador is the largest oxbow lake of the MBR, approximately 6.6 km long and 175 m wide, and constitutes the entirety of the Salvador otter family's territory.

### Data Collection

During 2004–2007, I observed the otters alone (2004–2006) or with one assistant (2007), amounting to 86, 45, 34, and 41 contact-hours, respectively during observation sessions of 3-h each at regular time periods (6–9AM, 9–12PM, 12–3PM, 3–6PM). Three hours was chosen as an observation session as it is approximately the time of duration of morning hunting bouts (typically beginning around 6AM); I assume in the analysis that each session provides an independent measure of catch and sharing activity. Contact-hours include time observing all activities other than resting in and around the den or campsites. I recorded helping behaviors using continuous sampling [50], following at close range from a kayak in all-day follows for periods of 1–2 weeks. The 2007 observations took place 11–19 September. Sampling occurred during dry season months (July–September) when newborn young typically appear. I tracked behavioral states and events on a palm pilot running customized event recorder software on a PocketC compiler [51]. Behavioral events that were recorded included: alarm, beg, fish catch, share, no share, and steal. The identity of individual otters responsible for each event was dictated into a voice recorder with date and time stamped to the second (Tungsten C Palm pilot running Audacity Audio Personal Software [52]), as was the size class (0–10cm, 10–20cm, 20–30cm, >30cm) and species of all fishes caught during observations. Additional behavioral observations such as leading the family's movements, grooming, playing, and defense (usually against caiman or the observer) were also dictated into the voice recorder.

### Data Classification and Analysis

Begging interactions can be long and complicated behaviors involving multiple beggars and also multiple potential donors when a large prey item is transferred more than once. For these analyses, a begging bout was counted only once for each potential donor, and then scored as resulting in a share, no share (refusal to share), or a steal. Once an item was shared or stolen, a new begging bout might ensue, focused on the new possessor (a new potential donor). I identified both the potential donor and the beggar whenever possible for each begging bout. Unknown donors were usually animals observed in open water that shared small items with young before re-submerging quickly, making identification difficult. If more than one animal begged simultaneously for the same prey item, the result for only the winning beggar was used, so as not to inflate the rate of no shares of any donor.

Many shares to a beggar are obvious donations of whole or partial prey items, initiated by the donor and clearly shared with the donor's intent. At times, however, separating steals from shares was difficult. A beggar typically approaches a family member in possession of a prey item, and perches on the shore or a nearby log, making typical begging vocalizations. Transfer of a prey item is commonly preceded by a period of defensive growling and avoidance of the beggar, and consumption by the donor of a large portion of the prey item. Most transferred items are eventually handed over without resistance; however, steals do occur, in which the beggar lunges toward the donor and snatches the prey item from the donor's mouth. Steals were recorded only rarely and only if the donor turned away as if to escape from the beggar just before the item was transferred. Steals were typically uncontested as I did not observe subsequent chases or retrieval of stolen prey.

I compare event counts in 2004–2006 (165 total contact hours in 143 sessions) with equivalent data from September 2007 (41 contact hours in 22 sessions), when the breeding female, Cacao, was first deemed to be in physical decline. For Figure 1, average counts of fish catches and shares for each 3-h observation session were calculated by age class, averaging the count of all animals present in that year in an age class for each 3-h session in each year. In 2007, each age class but the <1-y age class is represented by a single individual. Means and 95% confidence intervals of the average counts by session were produced using bootstrap resampling [53], [54]. Age class categories include young of the year (“<1 y”), 1-2 y (“1-y”), 2-3 y (“2-y”), 3-4 y (“3-y”), and the breeding male and female.

## Supporting Information

Figure S1 Cacao (right) waits for a share from her daughter Ziggy, September 13, 2007. Photo by Melisse Reichmann.
(7.22 MB TIF)

Figure S2 Giant otters on Cocha Salvador.
(7.66 MB TIF)

Video S1 Firecat shares with Cacao. Cacao receives a portion of a large corvina (*Plagioscion squamosissimus*) from Firecat. Cacao can be identified by the few spots on her chest and her pink left ear. Video by Lisa Davenport, edited for brevity.
(14.28 MB MP4)

## References

[1] de Waal FBM, Zak PJ (2007). How selfish an animal? The case of primate cooperation.. Free Enterprise: Values in Action Conference Series, 2005–2006, MORAL MARKETS: THE CRITICAL ROLE OF VALUES IN THE ECONOMY.

[2] Weir AAS, Chappell J, Kacelnik A (2002). Shaping of hooks in New Caledonian crows.. Science.

[3] Struhsaker TT, Leland L (1977). Palmnut smashing by *Cebus a. apella* in Colombia.. Biotropica.

[4] Rendell L, Whitehead H (2001). Culture in whales and dolphins.. Behav Brain Sci.

[5] van Schaik CP, Ancrenaz M, Borgen G, Galdikas B, Knott CD (2003). Orangutan cultures and the evolution of material culture.. Science.

[6] Whiten A, Goodall J, McGrew WC, Nishida T, Reynolds V (1999). Cultures in chimpanzees.. Nature.

[7] Brosnan SF, de Waal FBM (2003). Monkeys reject unequal pay.. Nature.

[8] Range F, Horn L, Viranyi Z, Huber L (2009). The absence of reward induces inequity aversion in dogs.. Proc Natl Acad of Sci.

[9] Wynne CDL (2004). Animal behaviour: fair refusal by capuchin monkeys.. Nature.

[10] Pavelka MSM, Fedigan LM (1991). Menopause: A comparative life history perspective.. Yearbook Physical Anthropol.

[11] McAuliffe K, Whitehead H (2005). Eusociality, menopause and information in matrilineal whales.. Trends Ecol Evol.

[12] Marsh H, Kasuya T (1986). Evidence for reproductive senescence in female cetaceans.. Rep Int Whal Commn (Special Issue.

[13] Douglas-Hamilton I, Bhalla S, Wittemyer G, Vollrath F (2006). Behavioural reactions of elephants towards a dying and deceased matriarch.. Appl Anim Behav Sci.

[14] Hart BL, Hart LA, Pinter-Wollman N (2008). Large brains and cognition: Where do elephants fit in?. Neuroscience & Biobehavioral Reviews.

[15] Malcolm J (1980). African wild dogs play every game by their own rules.. Smithsonian.

[16] Fossey D (1983). Gorillas in the mist.

[17] de Waal FBM (1997). Bonobo: The forgotten ape.

[18] Duplaix N (1980). Observations on the ecology and behavior of the giant river otter *Pteronura brasiliensis* in Suriname.. Rev Ecol (Terre et Vie).

[19] Davenport LC (2008). Behavior and ecology of the giant otter (Pteronura brasiliensis) in oxbow lakes of the Manu Biosphere Reserve, Peru [PhD Dissertation].

[20] Schenck C (1999). Lobo de Río (*Pteronura brasiliensis*): Presencia, uso del habitat y protección en el Perú [PhD Dissertation].

[21] Staib E (2001). Öko-ethologie von riesenottern (*Pteronura brasiliensis*) in Peru. [PhD Dissertation].

[22] Sykes-Gatz S (2005). International giant otter studbook husbandry and management information and guidelines (2005).

[23] Groenendijk J, personal communication

[24] Pieja GC, personal communication

[25] Goodall J (1986). The Chimpanzees of Gombe: Patterns of behavior.

[26] Harrington FH, Mech DL, Fritts SH (1983). Pack size and wolf pup survival: their relationship under varying ecological conditions.. Behav Ecol Sociobiol.

[27] Mech DL, Boitani L (2003). Wolves: Behavior, ecology and conservation.

[28] Hamilton WD (1964). The genetical evolution of social behaviour.. J Theoret Biol.

[29] Greve W, Kierdorf H, Kierdorf U (2009). The Akela-effect – Is there an evolutionary benefit from senators in mammals?. Biosci Hypothes.

[30] Carter SK, Rosas FCW (1997). Biology and conservation of the Giant Otter *Pteronura brasiliensis*.. Mammal Rev.

[31] McComb K, Moss C, Durant SM, Baker L, Sayialel S (2001). Matriarchs as repositories of social knowledge in African elephants.. Science.

[32] Foley CAH, Pettorelli N, Foley L (2008). Severe drought and calf survival in elephants.. Biol Lett.

[33] Bates LA, Lee PC, Njiraini N, Poole JH, Sayialel K, al et (2008). Do Elephants Show Empathy?. Journal of Consciousness Studies.

[34] Lee PC (1987). Allomothering among African elephants.. Anim Behav.

[35] Richardson DS, Burke T, Komdeur J, Wedell N (2007). Grandparent helpers: the adaptive significance of older, postdominant helpers in the Seychelles warbler.. Evolution.

[36] Hawkes K, O'Connell JF, Jones NGB, Alvarez H, Charnov EL (1998). Grandmothering, menopause, and the evolution of human life histories.. Proc Natl Acad of Sci.

[37] Diamond J (1997). Why is sex fun? The evolution of human sexuality.

[38] Hill K, Hurtado AM (1991). The evolution of premature reproductive senescence and menopause in human females: An evaluation of the “Grandmother Hypothesis”.. Hum Nat.

[39] Packer C (1998). Reproductive cessation in female mammals.. Nature.

[40] Groenendijk J, Hajek F (2006). Giants of the Madre de Dios.

[41] Corredor GL, Tigreros NM (2006). Reproduction, behaviour and biology of the Giant River Otter (*Pteronura brasiliensis*) at Cali Zoo.. Intl Zoo Yearbook.

[42] Corredor GL, personal communication

[43] Sharp SP, Clutton-Brock TH (2010). Reproductive senescence in a cooperatively breeding mammal.. J Anim Ecol:.

[44] Animal Behavior Society (2007). ABS/ABAS Guidelines for the treatment of animals in behavioural research and teaching.. Animal Behavior Society Handbook.

[45] Gannon WL, Sikes RS, Animal Care and Use Committee of the American Society of Mammalogists (2007). Guidelines of the American Society of Mammalogists for the use of wild mammals in research.. J Mamm.

[46] Gentry AH, Terborgh JW, Gentry AH (1990). Composition and dynamics of the Cocha Cashu mature floodplain forest, Peru.. Four Neotropical Rainforests.

[47] Terborgh JW (1983). Five New World Primates: A study in comparative ecology.

[48] Kalliola R, Puhakka M, Kalliola R, Puhakka M, Danjoy W (1993). Geografia de la selva baja Peruana.. Amazonia Peruana: Vegetación húmeda tropical en el llano subandino.

[49] Goulding M, Cañas C, Barthem R, Forsberg B, Ortega H (2003). Amazon Headwaters: rivers, wildlife, and conservation in south-eastern Peru.

[50] Martin P, Bateson P (1993). Measuring behaviour: An introductory guide.

[51] Lorch P (2002). Event Recorder 1.3: Palm pilot freeware.. http://www.pitt.edu/~lorch/EveRec.html.

[52] Audacity Audio (2002). – Personal Voice Recorder Software for Palm OS.. http://www.audacityaudio.com.

[53] Manly BFJ (1997). Randomization, bootstrap, and Monte Carlo methods in biology.

[54] Statistics.com LLC (2009). Resampling statistics add-in for Excel 2003, v. 4.. http://www.resample.com.

