# Supplementary figures and images for: Aid to a Declining Matriarch in the Giant Otter (Pteronura brasiliensis)
Source: PLoS One. 2010 Jun 30;5(6):e11385. doi: 10.1371/journal.pone.0011385 (PMC2894880; doi:10.1371/journal.pone.0011385)

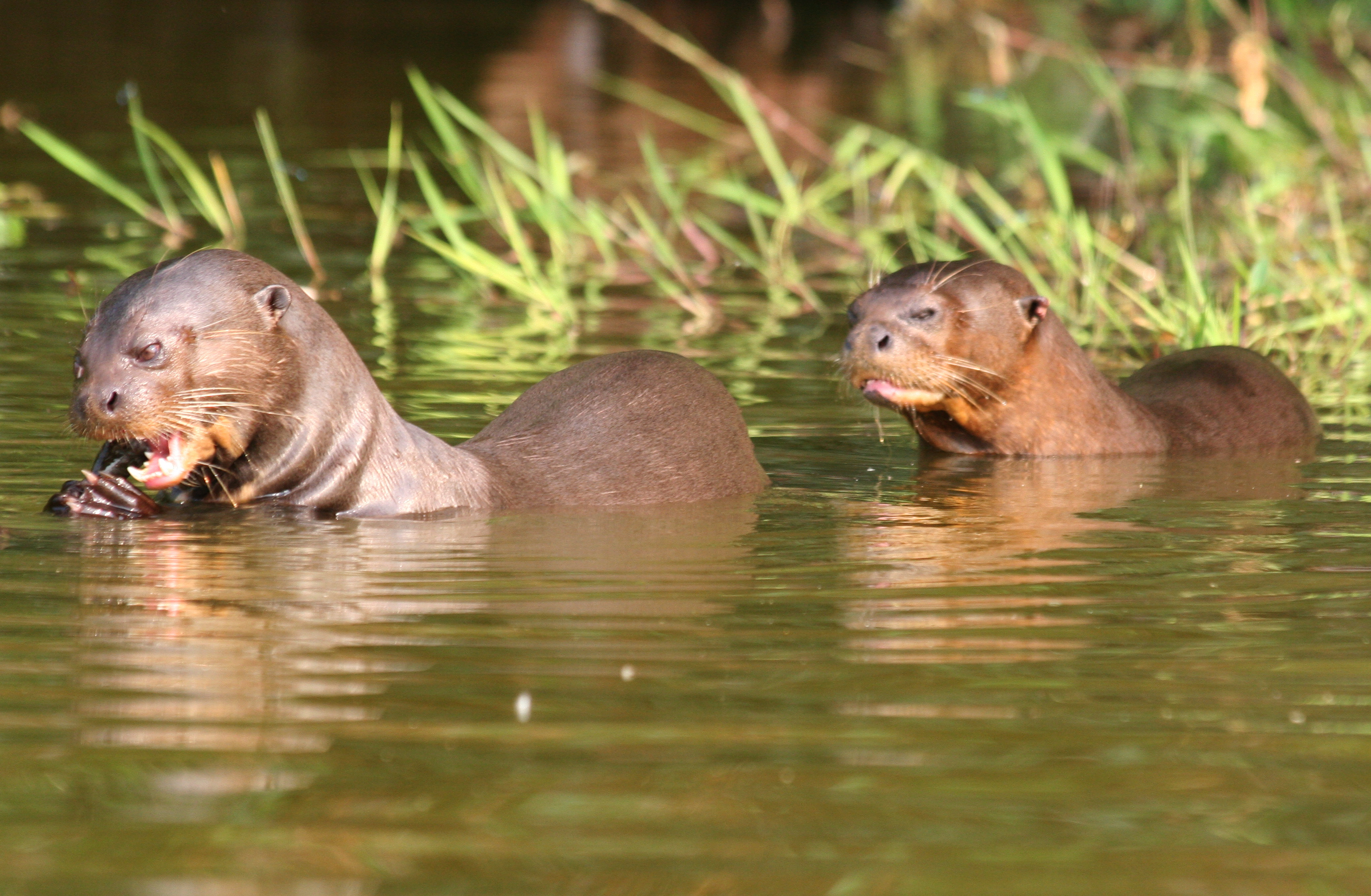

Supplement: Figure S1 — Cacao (right) waits for a share from her daughter Ziggy, September 13, 2007. Photo by Melisse Reichmann. (7.22 MB TIF) [file pone.0011385.s001.tif]

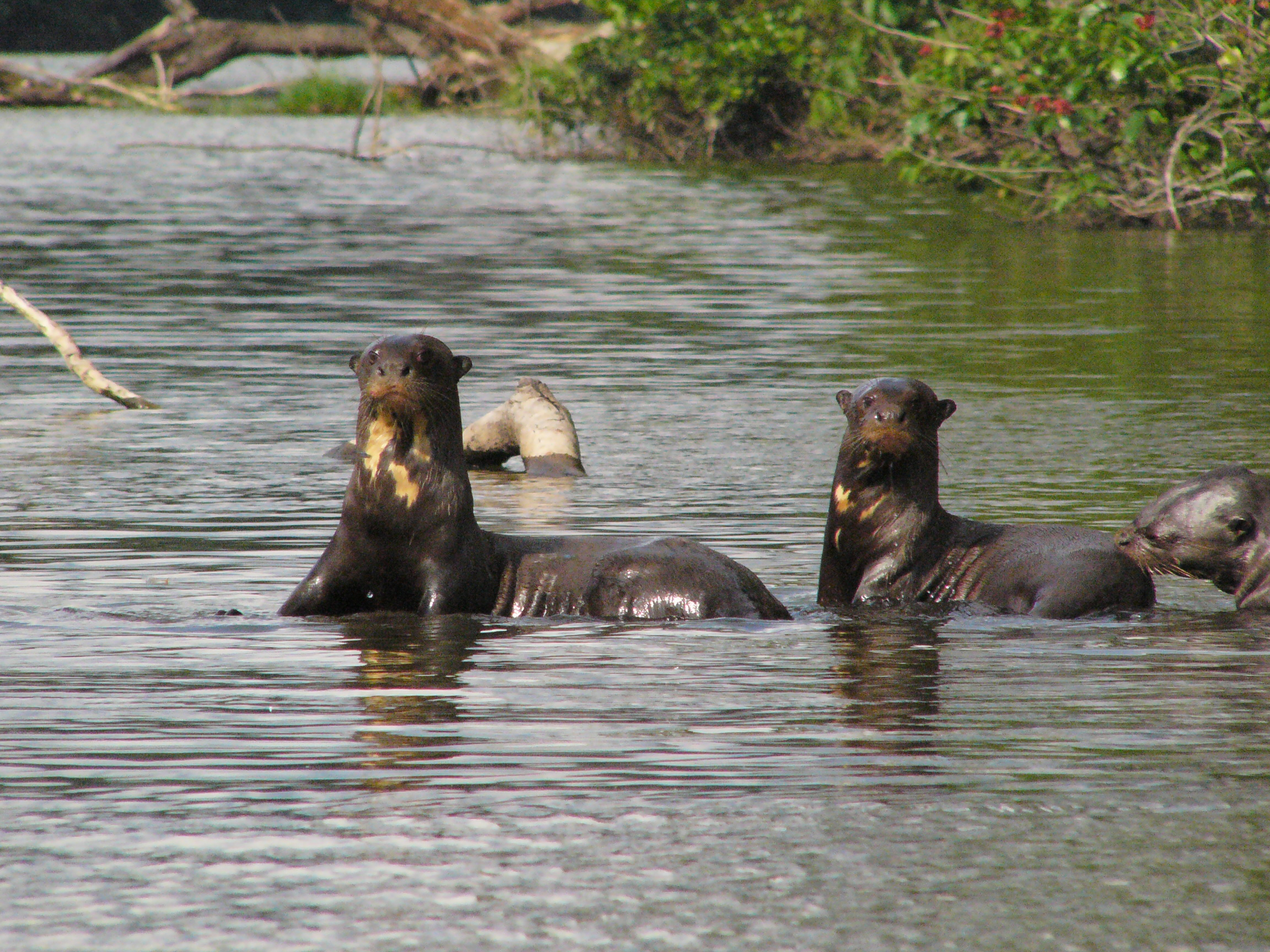

Supplement: Figure S2 — Giant otters on Cocha Salvador. (7.66 MB TIF) [file pone.0011385.s002.tif]
